# Real-time multiplex PCR for human echinococcosis and differential diagnosis[Fn FN1]

**DOI:** 10.1051/parasite/2023003

**Published:** 2023-01-25

**Authors:** Jenny Knapp, Séverine Lallemand, Franck Monnien, Sophie Felix, Sandra Courquet, Gérald Umhang, Laurence Millon

**Affiliations:** 1 Department of Parasitology-Mycology, National Reference Centre for Echinococcoses, University Hospital of Besançon 25030 Besançon France; 2 UMR CNRS 6249 Laboratoire Chrono-environnement, University of Franche-Comté 16 Route de Gray 25030 Besançon France; 3 Department of Pathology, University Hospital of Besançon 25030 Besançon France; 4 ANSES Nancy laboratory for Rabies and Wildlife, National Reference Laboratory for Echinococcus spp., Wildlife Surveillance and Eco-epidemiology Unit, Technopole Agricole et Vétérinaire 54220 Malzéville France

**Keywords:** Human echinococcosis, Molecular diagnosis, Fresh material, FFPE, Quantitative real-time PCR, Multiplexing

## Abstract

Molecular identification of rare human infectious pathogens appears to be one of the most relevant current methods for rapid diagnosis and management of patients. PCR techniques, in particular real-time quantitative PCR, are best suited for the detection of DNA from the pathogens, even at low concentrations. Echinococcosis infections are due to helminths of the *Echinococcus* genus, with closely related species involved in parasitic lesions affecting animals and, accidentally, humans. We developed a multiplex qPCR (MLX qPCR) assay allowing for the detection of four *Echinococcus* species involved in Europe in alveolar echinococcosis (AE) and cystic echinococcosis (CE) (*Echinococcus multilocularis*, *E. granulosus sensu stricto*, *E. ortleppi*, and *E. canadensis*), based on short mitochondrial targets. A collection of 81 fresh and formalin-fixed paraffin-embedded tissues (FFPE) of AE and CE lesions was assembled. The qPCR assays were performed in triplex for *Echinococcus* spp. detection, associated with a qPCR inhibitor control. A duplex qPCR was also designed to enable diagnosis of two other dead-end helminthiases (cysticercosis (*Taenia solium*), and toxocariasis (*Toxocara cati* and *T. canis*)). The sensitivity of the qPCR was assessed and ranged from 1 to 5 × 10^−4^ ng/μL (seven PCR assays positive), corresponding to 37–42 cycles for quantifiable DNA. The specificity was 100% for all the targets. This multiplex qPCR, adapted to low amounts of DNA can be implemented in the laboratory for the rapid molecular diagnosis of *Echinococcosis* species.

## Abstract (fr)

L’identification moléculaire des pathogènes infectieux humains rares semble être l’une des méthodes actuelles les plus pertinentes pour un diagnostic et une prise en charge rapides des patients. Les techniques de PCR, en particulier la PCR quantitative en temps réel, sont bien adaptées à la détection d’ADN de pathogènes, même pour de faibles concentrations. Les infections à échinocoque sont dues à des helminthes du genre *Echinococcus*, des espèces étroitement apparentées, impliquées dans des lésions parasitaires affectant les animaux et accidentellement l’homme. Une qPCR multiplex (MLX qPCR), permettant la détection de quatre espèces d’*Echinococcus* impliquées en Europe dans l’échinococcose alvéolaire (EA) et kystique (EK) (*Echinococcus multilocularis*, *E. granulosus sensu stricto*, *E. ortleppi* et *E. canadensis*), basée sur de courtes cibles mitochondriales a été développée ici. Une collection a été constituée de 81 tissus frais ou fixés en paraffine (FFPE) de lésions d’EA et EK. Les essais de qPCR ont été réalisées en triplex pour la détection d’*Echinococcus* spp., associés à une qPCR de contrôle d’inhibition. Une PCR duplex a été développée pour le diagnostic de deux autres helminthiases en impasse chez l’Homme (cysticercose (*Taenia solium*), et toxocarose (*Toxocara cati* et *T. canis*). La sensibilité de la qPCR a été évaluée et s’échelonne de 1 à 5 × 10^−4^ ng/μl (sept essais de qPCR positifs), correspondant à 37 à 42 cycles pour l’ADN quantifiable. La spécificité était de 100 % pour toutes les cibles. Cette qPCR multiplex, adaptée à de faibles quantités d’ADN peut être mise en œuvre au laboratoire pour un diagnostic moléculaire rapide des espèces d’*Echinococcus*.

## Introduction

In medical biology, molecular diagnosis based on non-cultivable endoparasites is an accurate method to confirm an infection for a suggestive clinical and epidemiological presentation due to its sensitivity and specificity, and because DNA is an unforgeable carrier of identity. Molecular diagnosis is particularly relevant in case of dead-end helminthiases, such as echinococcosis, toxocariasis, or cysticercosis. Indeed, no exit of eggs or adult stages is observed by direct examination. In such cases, besides imaging which is often the first step in the diagnosis, detection of specific antibodies is the main diagnosis tool, but this type of approach is subject to problems concerning specificity and sensitivity. Moreover, serological assays can lead to negative results for immunosuppressed patients because of their immune status [6, 7]. In this context, molecular diagnosis provides incontrovertible species identification on surgery or needle biopsy samples.

Alveolar and cystic echinococcosis are two serious diseases caused by cestodes from the genus *Echinococcus* Rudolphi (1801), often with years between the first contact with the parasite and diagnosis. Human alveolar echinococcosis (AE) is due to *Echinococcus multilocularis* Leuckart (1863), whereas cystic echinococcosis (CE) is caused by several species, grouped under the taxon name *Echinococcus granulosus* sensu lato (s.l.) [40], known to circulate in Europe, with human cases reported [5]. In Europe, the complex comprises *E. granulosus sensu stricto* (s.s.) Batsch (1786), first described in sheep as an intermediate host, *E. canadensis* (Webster and Cameron, 1961), described in camels (previous G6), pigs (G7), and deer (G8, G10), depending on the geographical occurrence, and *E. ortleppi* Lopez-Neyra and Soler Planas (1943), described in cattle. *Echinococcus multilocularis* is endemic only in the northern hemisphere and essentially maintained by a sylvatic lifecycle between red foxes and small mammals in Europe, whereas *E. granulosus* s.l. has a worldwide distribution, mainly supported by a domestic lifecycle between dogs and livestock. CE is classified as the second and AE as the third most relevant food-borne parasitic diseases in the world [11]. However, due to its greater mortality, AE is classified as the most severe helminthic zoonosis in the northern hemisphere [34, 35]. The diagnoses of human AE and CE require multidisciplinary consensus before implementing chemical treatment and surgery, when possible, which is the only curative treatment for echinococcosis infections. AE occurs mostly in the liver and CE in the liver and lungs [33]. For molecular diagnosis in humans, various matrices can be analysed, such as tissues from various organs, liquid punctures, and formalin-fixed paraffin-embedded (FFPE) tissues. In addition to *Echinococcus* spp. infections, molecular diagnosis could help in the diagnosis of “dead-end” helminthiases. Toxocariasis, a worldwide parasitosis, can also involve the same organs as *Echinococcus* spp., especially the liver and the brain. In humans, this disease is due to the ingestion of embryonated eggs of *Toxocara cati*, a nematode of cats, or *T. canis*, a nematode of dogs and foxes [8]. Humans are an aberrant host for *Toxocara* spp. because of the non-development of the L3 larvae stage in adults, as in dogs and cats, resulting in *larva migrans*. In patients, the liver is the most frequent organ involved, with abscess presentation [16]. Human cysticercosis is a parasitosis due to the tapeworm *Taenia solium*, with the larval stage described in humans in muscles, the brain, the eyes, or, indeed, any organ, and is still a problem in a number of farming communities in developing countries of Africa, Asia, and Latin America [29]. Humans act as an intermediate host after the ingestion of tapeworm eggs present on contaminated vegetables or by self-contamination due to the faecal-oral cycle (cysticercus larva).

Quantitative PCR (qPCR) is a promising tool to detect and quantify DNA from infectious agents in different specimens (tissue, fluids, blood) and has allowed a step forward in the diagnosis of many infectious diseases, and especially parasitic diseases in the last decade [15, 31]. The advantages of qPCR-based techniques are higher sensitivity than end-point PCR, a reduction in PCR product contamination because of the limited manipulation of amplicons, and the possibility to quantify the targeted DNA. In addition, multiplexing in qPCR, permitted by multiple fluorescent channel dyed-amplicon detection, allows the detection of large panels of targets using the same DNA extract. Multiplex qPCR can be used in screening for first-line molecular diagnosis, with reduction in the quantity of DNA and PCR reagents required, and a reduced handling time.

For detection of the parasite *Echinococcu*s sp., several qPCR assays have been developed. Some of them are able to detect the parasite *E. multilocularis* and hosts in environmental samples [9, 21]. Other qPCR assays are able to detect co-occurrence of parasites (for example *E. multilocularis* and *E. canadensis* in definitive hosts [30,43], *E. multilocularis* and *Toxoplasma gondii* [32], or *Toxocara* spp. [21] in environmental samples. Some qPCR assays were also developed for the diagnosis of echinococcosis in humans (*E. multilocularis* and *E. granulosus* s.l. in tissue sample [4] or in plasma [24]). However, none of the published qPCR assays is able to detect all the species potentially involved in human echinococcosis from tissue samples, and especially from FFPE specimens.

Here, we aimed to develop and assess a test for the accurate and sensitive molecular diagnosis of echinococcosis in humans from tissue samples. We developed a multiplex real-time quantitative PCR assay (MLX qPCR) targeting the four species that can be encountered in Europe (*E. multilocular*is, *E. granulosus* ss, *E. canadensis*, and *E. ortleppi*), combined with an external control for the detection of inhibitors. The MLX qPCR was also designed to offer diagnosis for human toxocariasis and cysticercosis, which could help to avoid diagnostic wandering in certain atypical clinical situations.

## Materials and methods

### Sample panel

A panel of 81 samples was established. The panel was composed of 46 AE (15 frozen tissues, 31 FFPE specimens) and 35 CE lesions (19 frozen tissues, 16 FFPE specimens), collected from 1997 to 2021, with the geographical origin of the patients when available (Table 1). The *Echinococcus* species of the sample material have been confirmed by either species-specific PCRs, qPCR or via sequencing in previous studies [19] (Table 1). The species of the AE lesions were identified as *E. multilocularis* and those of the CE samples as *E. granulosus* s.s. (*n* = 29), *E. canadensis* (*n* = 4), and *E. ortleppi* (*n* = 2). For frozen tissues, DNA was purified using a High Pure PCR Template Preparation kit (Roche Diagnostics, Mannheim, Germany) from cubic millimetre pieces of the surgical sample, following the manufacturer’s protocol, and the DNA eluted in a volume of 200 μL of the provided buffer. For FFPE samples, DNA was purified and extracted immediately after de-paraffining the FFPE shavings using a QIAamp DNA FFPE Tissue kit (Qiagen, Hilden, Germany), following the manufacturer’s protocol, and the DNA eluted in a volume of 50 μL of the provided buffer. All DNA extracts were stored at −20 °C until use. The DNA concentration from total DNA extracts was determined from 2 μL using a nanophotometer apparatus (Implen, Munich, Germany).

**Table 1:** Collection of alveolar and cystic echinococcosis lesion samples stored fresh or frozen or as formalin-fixed paraffin-embedded (FFPE) samples and qPCR results (Cq value and DNA concentration assessment) for the samples.

Sample code	Geographical origin of patient	Disease	Species	Year of sampling	Tissue type	Lesion location/origin	MLX qPCR tested	Alea	rrn	Em	Eg	Ec	Eo	Tsol	Toxo	DNA quantif (ng/μL)
								(qPCR results in mean cycle number)								
FE-577	France	AE	*Em*	2021	FFPE	Liver	1-2-3	33.4	ND	27.5	*>45*	*>45*	*>45*	*>45*	*>45*	0.9
FE-842	France	AE	*Em*	2021	Frozen	Liver	1	32.6	ND	25.1	*>45*	ND	ND	ND	ND	5.0
FE-875	France	AE	*Em*	2021	Frozen	Liver	1-2-3	31.3	ND	25.5	*>45*	*>45*	*>45*	*>45*	*>45*	3.9
FE-878	France	AE	*Em*	2021	Frozen	Liver	1-2-3	34.3	ND	24.9	*>45*	*>45*	*>45*	*>45*	*>45*	4.6
FE-907	France	AE	*Em*	2021	Frozen	Liver	1-2-3	32.7	ND	23.9	*>45*	*>45*	*>45*	*>45*	*>45*	12.4
FE-917	France	AE	*Em*	2021	Frozen	Peritoneal	1-2-3	31.6	ND	36.0	*>45*	*>45*	*>45*	*>45*	*>45*	0.002
FE-929	France	AE	*Em*	2021	FFPE	Liver	1-2-3	32.9	ND	33.4	*>45*	*>45*	*>45*	*>45*	*>45*	0.01
LU-O	Luxemburg	AE	*Em*	2021	Frozen	Liver	1-2-3	32.3	ND	27.5	*>45*	*>45*	*>45*	*>45*	*>45*	0.9
FE-755	France	AE	*Em*	2020	FFPE	Peri-duodenal	1-2-3	34.3	ND	29.0	*>45*	*>45*	*>45*	*>45*	*>45*	0.3
FE-861	France	AE	*Em*	2020	Frozen	Liver	1-2-3	31.6	ND	27.1	*>45*	*>45*	*>45*	*>45*	*>45*	1.2
FE-908	France	AE	*Em*	2020	FFPE	Brain	1-2-3	34.2	ND	25.8	*>45*	*>45*	*>45*	*>45*	*>45*	3.0
FE-918	France	AE	*Em*	2020	FFPE	Liver	1-2-3	34.8	ND	36.7	*>45*	*>45*	*>45*	*>45*	*>45*	0.001
FE-836	France	AE	*Em*	2019	Frozen	Liver	1-2-3	34.2	ND	31.2	*>45*	*>45*	*>45*	*>45*	*>45*	0.1
FE-652	France	AE	*Em*	2018	Frozen	Liver	1-2-3	33.7	ND	29.4	*>45*	*>45*	*>45*	*>45*	*>45*	0.2
FE-653	France	AE	*Em*	2018	Frozen	Liver	1-2-3	33.8	ND	25.5	*>45*	*>45*	*>45*	*>45*	*>45*	3.7
FE-766	France	AE	*Em*	2018	Frozen	Foie	1-2-3	34.2	ND	26.5	*>45*	*>45*	*>45*	*>45*	*>45*	1.8
FE-792	France	AE	*Em*	2018	Frozen	Liver	1-2-3	33.8	ND	26.5	*>45*	*>45*	*>45*	*>45*	*>45*	1.8
FE-800	France	AE	*Em*	2018	Frozen	Spleen	1-2-3	34.0	ND	25.9	*>45*	*>45*	*>45*	*>45*	*>45*	3.0
FE-805	France	AE	*Em*	2018	Frozen	Brain	1-2-3	34.2	ND	23.7	*>45*	*>45*	*>45*	*>45*	*>45*	14.5
FE-806	France	AE	*Em*	2018	FFPE	Adrenal	1-2-3	34.7	ND	22.9	*>45*	*>45*	*>45*	*>45*	*>45*	25.0
FE-829	France	AE	*Em*	2018	Frozen	Liver	1-2-3	33.0	ND	26.2	*>45*	*>45*	*>45*	*>45*	*>45*	2.2
FE-768	France	AE	*Em*	2017	FFPE	Peritoneal	1	34.5	ND	28.4	*>45*	ND	ND	ND	ND	0.5
FE-782	France	AE	*Em*	2017	FFPE	Liver	1	36.4	ND	22.9	*>45*	ND	ND	ND	ND	26.2
P16-1535	France	AE	*Em*	2016	FFPE	Liver	1	34.5	35.5	30.9	*>45*	ND	ND	ND	ND	0.08
P16-2314	France	AE	*Em*	2016	FFPE	Liver	1	35.0	39.2	36.3	*>45*	ND	ND	ND	ND	0.002
P16-5377	France	AE	*Em*	2016	FFPE	Liver	1	34.9	29.3	29.3	*>45*	ND	ND	ND	ND	0.25
FE-769	France	AE	*Em*	2015	FFPE	Liver	1	34.5	ND	28.9	*>45*	ND	ND	ND	ND	0.3
P15-11460	France	AE	*Em*	2015	FFPE	Liver	1	35.4	40.8	39.2	*>45*	ND	ND	ND	ND	0.0002
P1515434.50	France	AE	*Em*	2015	FFPE	Liver	1	34.8	31.7	31.9	*>45*	ND	ND	ND	ND	0.04
P15-15434	France	AE	*Em*	2015	FFPE	Liver	1	35.4	38.9	34.8	*>45*	ND	ND	ND	ND	0.005
P14-3252	France	AE	*Em*	2014	FFPE	Liver	1	35.1	36.1	31.9	*>45*	ND	ND	ND	ND	0.04
P14-8880	France	AE	*Em*	2014	FFPE	Liver	1	35.0	31.5	31.5	*>45*	ND	ND	ND	ND	0.05
P1315022.50	France	AE	*Em*	2013	FFPE	Liver	1	34.8	27.4	27.3	*>45*	ND	ND	ND	ND	1.05
P13-4653IIC	France	AE	*Em*	2013	FFPE	Liver	1-2	35.1	41.1	*>45*	*>45*	*>45*	*>45*	ND	ND	*>45*
FE-904	Poland	AE	*Em*	2012	FFPE	Liver	1-2-3	34.2	ND	29.7	*>45*	*>45*	*>45*	*>45*	*>45*	0.2
P12-16504ES	France	AE	*Em*	2012	FFPE	Liver	1	34.9	38.4	34.6	*>45*	ND	ND	ND	ND	0.01
P1215706III	France	AE	*Em*	2012	FFPE	Liver	1	34.8	36.4	32.9	*>45*	ND	ND	ND	ND	0.02
P11-12405	France	AE	*Em*	2011	FFPE	Liver	1	34.9	40.8	*>45*	*>45*	ND	ND	ND	ND	*>45*
P1112967.50	France	AE	*Em*	2011	FFPE	Liver	1-2	35.1	35.0	35.8	*>45*	*>45*	*>45*	ND	ND	0.002
P08-1905I	France	AE	*Em*	2008	FFPE	Liver	1	34.9	38.6	35.5	*>45*	ND	ND	ND	ND	0.003
P06-11605I	France	AE	*Em*	2006	FFPE	Liver	1	34.9	35.9	38.7	*>45*	ND	ND	ND	ND	0.0003
P06-4509III	France	AE	*Em*	2006	FFPE	Liver	1	34.9	35.6	34.6	*>45*	ND	ND	ND	ND	0.01
P05-14960II	France	AE	*Em*	2005	FFPE	Liver	1	35.1	33.9	33.2	*>45*	ND	ND	ND	ND	0.01
S00-15720II	France	AE	*Em*	2000	FFPE	Sub cutan.	1	35.2	32.2	31.9	*>45*	ND	ND	ND	ND	0.04
S99-6011IIA	France	AE	*Em*	1999	FFPE	Liver	1	34.7	36.3	39.0	*>45*	ND	ND	ND	ND	0.0002
S97-9110IV	France	AE	*Em*	1997	FFPE	Liver	1	35.0	28.0	27.9	*>45*	ND	ND	ND	ND	0.68
EK-A20	Morocco	CE	*Eg*	2021	frozen	Liver	1-2-3	31.6	ND	*>45*	21.7	*>45*	*>45*	*>45*	*>45*	139.5
EK-B21	ND	CE	*Eg*	2021	FFPE	Liver	1-2-3	34.4	ND	*>45*	37.1	*>45*	*>45*	*>45*	*>45*	0.003
EK-B22	France	CE	*Eg*	2021	FFPE	Lung	1-2-3	32.7	ND	*>45*	39.0	*>45*	*>45*	*>45*	*>45*	0.001
EK-G23	France	CE	*Eg*	2021	FFPE	Lung	1	38.4#	ND	*>45*	27.1	ND	ND	ND	ND	3.3
EK-G24	Romania	CE	*Eg*	2021	Frozen	Liver	1-2-3	32.9	ND	*>45*	25.4	*>45*	*>45*	*>45*	*>45*	10.4
EK-K25	Turkey	CE	*Eg*	2021	Frozen	Liver	1-2-3	32.9	ND	*>45*	22.3	*>45*	*>45*	*>45*	*>45*	94.6
EK-H13	Algeria	CE	*Eg*	2020	Frozen	Liver	1-2-3	33.8**	ND	*>45*	23.4	*>45*	*>45*	*>45*	*>45*	42.6
EK-L14	Morocco	CE	*Eg*	2020	FFPE	Intra-abdominal	1-2-3	31.6	ND	*>45*	36.6	*>45*	*>45*	*>45*	*>45*	0.004
EK-L15	Morocco	CE	*Eg*	2020	Frozen	Kidney	1-2-3	31.9	ND	*>45*	22.3	*>45*	*>45*	*>45*	*>45*	94.2
EK-M16	Algeria	CE	*Eg*	2020	FFPE	Liver	1-2-3	33.5	ND	*>45*	35.8	*>45*	*>45*	*>45*	*>45*	0.01
EK-M17	Morocco	CE	*Eg*	2020	Frozen	Bottom	1-2-3	34.2**	ND	*>45*	25.6	*>45*	*>45*	*>45*	*>45*	9.0
EK-M18	Morocco	CE	*Eg*	2020	FFPE	Bone	1	34.8	ND	*>45*	33.0	ND	ND	ND	ND	0.05
EK-S19	France	CE	*Eg*	2020	Frozen	Spleen	1-2-3	34.2**	ND	*>45*	21.0	*>45*	*>45*	*>45*	*>45*	239.9
EK-M11	Tunisia	CE	*Eg*	2019	Frozen	Lung	1-2-3	*>45*	ND	*>45*	16.0	*>45*	*>45*	*>45*	*>45*	8300.4
EK-M12	Tunisia	CE	*Eg*	2019	Frozen	Lung	1-2-3	34.1	ND	*>45*	21.8	*>45*	*>45*	*>45*	*>45*	134.1
EK-D28	Mali	CE	*Ec*	2019	Frozen	Lung	1-2-3	32.2	ND	*>45*	*>45*	14.7	*>45*	*>45*	*>45*	2617.2
EK-H29	ND	CE	*Ec*	2019	Frozen	Lung	1-2-3	33.2	ND	*>45*	*>45*	28.2	*>45*	*>45*	*>45*	0.2
EK-B1	Algeria	CE	*Eg*	2018	FFPE	Liver	1-2-3	34.7	ND	*>45*	27.6	*>45*	*>45*	*>45*	*>45*	2.3
EK-C2	Moldova	CE	*Eg*	2018	Frozen	Bone	1-2-3	33.9	ND	*>45*	33.2	*>45*	*>45*	*>45*	*>45*	0.04
EK-C3	Moldova	CE	*Eg*	2018	Frozen	Bone	1-2-3	33.3	ND	*>45*	32.8	*>45*	*>45*	*>45*	*>45*	0.06
EK-H4	France	CE	*Eg*	2018	FFPE	NR	1-2-3	34.4	ND	*>45*	32.9	*>45*	*>45*	*>45*	*>45*	0.05
EK-H5	France	CE	*Eg*	2018	FFPE	NR	1-2-3	34.1	ND	*>45*	35.9	*>45*	*>45*	*>45*	*>45*	0.01
EK-K6	Morocco	CE	*Eg*	2018	Frozen	Bedsore	1-2-3	33.9	ND	*>45*	31.9	*>45*	*>45*	*>45*	*>45*	0.1
EK-K7	Morocco	CE	*Eg*	2018	Frozen	Bedsore	1-2-3	34.2	ND	*>45*	31.7	*>45*	*>45*	*>45*	*>45*	0.1
EK-L8	France	CE	*Eg*	2018	FFPE	Lung	1-2-3	34.9	ND	*>45*	37.9	*>45*	*>45*	*>45*	*>45*	0.002
EK-M9	Moldova	CE	*Eg*	2018	Frozen	Intraperitoneal	1-2-3	34.1	ND	*>45*	32.1	*>45*	*>45*	*>45*	*>45*	0.09
EK-T10	Tibet	CE	*Eg*	2018	Frozen	NR	1-2-3	33.9	ND	*>45*	24.9	*>45*	*>45*	*>45*	*>45*	15.6
EK-S27	Mauritania	CE	*Ec*	2018	Frozen	NR	1-2-3	31.9	ND	*>45*	*>45*	26.9	*>45*	*>45*	*>45*	0.6
EK-S26	ND	CE	*Ec*	2017	Frozen	Lung	1-2-3	37.5	ND	*>45*	*>45*	21.0	*>45*	*>45*	*>45*	34.2
EK-M30*	France	CE	*Eo*	2017	FFPE	Bone	1-2-3	34.4	ND	*>45*	*>45*	*>45*	34.7	*>45*	*>45*	0.03
EK-R31*	France	CE	*Eo*	2017	FFPE	Bone	1-2-3	34.2	ND	*>45*	*>45*	*>45*	25.8	*>45*	*>45*	59.4
P10-8854†,$	Turkey	CE	*Eg*	2010	FFPE	NA	1-2	35.2	*>45*	*>45*	39.9#	*>45*	*>45*	ND	ND	0.0004
P06-5181.50	ND	CE	*Eg*	2006	FFPE	NA	1-2	35.4	*>45*	*>45*	38.8	*>45*	*>45*	ND	ND	0.001
P0517231.50	ND	CE	*Eg*	2005	FFPE	Liver	1-2	35.1	*>45*	*>45*	36.9	*>45*	*>45*	ND	ND	0.003
P05-17231	ND	CE	*Eg*	2005	FFPE	Liver	1-2	35.4	*>45*	*>45*	37.0	*>45*	*>45*	ND	ND	0.003
Em	France	Control	*Em*	2005	Frozen	Fox	1-2-3	35.1	ND	22.9	*>45*	*>45*	*>45*	*>45*	*>45*	25.8
5872	France	Control	*Eg*	2012	Frozen	Ovine	1-2-3	33.0	ND	*>45*	24.7	*>45*	*>45*	*>45*	*>45*	17.5
3734	France	Control	*Ec*	2010	Frozen	Pig	1-2-3	34.7	ND	*>45*	*>45*	23.2	*>45*	*>45*	*>45*	8.0
6697	France	Control	*Eo*	2012	Frozen	Bovine	1-2-3	34.1	ND	*>45*	*>45*	*>45*	23.8	*>45*	*>45*	337.5
Ts-Pasteur	France	Control	*Tsol*	ND	Frozen	ND	1-2-3	34.8	ND	*>45*	*>45*	*>45*	*>45*	15.9	*>45*	86.2
Tcati	France	Control	*Tcat*	ND	Frozen	Cat	1-2-3	34.3	ND	*>45*	*>45*	*>45*	*>45*	*>45*	24.5	0.4
Tcanis	France	Control	*Tcan*	ND	Frozen	Fox	1-2-3	34.8	ND	*>45*	*>45*	*>45*	*>45*	*>45*	20.8	1.9

AE, alveolar echinococcosis; CE, cystic echinococcosis; rrn, qPCR targeting fragment of the mitochondrial 16S-*rrnL* gene [20]; Em, *Echinococcus multilocularis*; Eg, *E. granulosus*; Ec, *E. canadensis*; Eo, *E. ortleppi*; Tsol, *Taenia solium*; Tcat, *Toxocara cati*; Tcan, *T. canis*. qPCR tested: T for 3 multiplex qPCR tested on the sample, qPCR MLX 1, Em–Eg–Alea, 2, Ec–Eo–Alea, 3, Tsol–Toxo.

ND for no data.

* Sample tested on 1:100 dilution.

** Alea qPCR negative on the triplex Em/Eg s.s./Alea and positive on the triplex Ec/Eo/Alea.

# qPCR positive once from the duplicate.

† Sample not described in Knapp et al. [19] but tested with the same PCR techniques.

$ Sample positive for the multiplex PCR from Trachsel et al. [37].

.50 For elution volume in 50 μL of elution buffer instead of 200 μL for the other FFPE samples.

### Real-time quantitative PCR design for *Echinococcus* spp. DNA detection and differential diagnosis

Four pairs of primers and associated TaqMan probes were designed for the present study to detect human echinococcosis from surgical specimens: *E. multilocularis*, *E. granulosus* s.s., *E. canadensis*, and *E. ortleppi*. The TaqMan hydrolysis probes and primers were designed from mitochondrial sequence data available in the NCBI genetic database for each species (Table 2) using Primer Express software v3.0 (Applied Biosystems) and were designed to specifically amplify the sequences targeted by the qPCR. For *E. canadensis*, primers and probe were designed from G6 and G7 genomes, in order to amplify the species *E. canadensis*. In addition, an external qPCR control was used, called the Alea target and designed in a previous study, to test the presence of PCR inhibitors [21].

**Table 2:** Primers and hydrolysis probes designed based on GenBank reference sequences for the targeted parasite detection by qPCR.

Target species	Target gene	Primer and probe	GenBank reference No (country of origin of the genotyped specimen)	Oligonucleotide sequence (5^′^–3^′^)	Probe label	Target size (bp)	References
*Echinococcus multilocularis*	*Cytochrome b*	Em_cytb_Fwd	AB018440 (Japan) [28]	CGAAAATCCACCAACCACATACT		84	This study
		Em_cytb_Rev		GCTGCCACTGTCCTTACTTCAA			
		Em_cytb_Probe		ACCATAGAACCAACCAACGGCAAACTATCA	FAM-TAMRA		
*Echinococcus granulosus s.s.*	*Cytochrome oxidase* subunit III	Eg_cox3_Fwd	AF297617 (for G1) (United Kingdom) [22]	TATCTGTAACACCACAAAACTCAAACC		149	This study
		Eg_cox3_Rev		CGTTGGAGATTCCGTTTGTTG			
		Eg_cox3_Probe		AACAAAAGCAAATCACAACAACGTCAACCC	CY5-TAMRA		
*Echinococcus canadensis*	*NADH dehydrogenase* subunit V	Ec_nad5_Fwd	AB208063 (for G6) (Kazakhstan) [26]	ATACGCCATGACTTATCAACTGAAAT		105	This study
		Ec_nad5_Rev	AB235847 (for G7) (Poland) [26]	AGATTGTGGCTTTGTCAACTTGTAA			
		Ec_nad5_Probe		TCACCAAAAATCAAGTACAAAACGCACCAA	FAM-TAMRA		
*Echinococcus ortleppi*	*ATP synthase* membrane subunit VI	Eo_atp6_Fwd	AB235846 (Argentina) [26]	CTTTTTAGTGTGTATAGCTGAGTCCATTAGT		145	This study
		Eo_atp6_Rev		CAACGCCCACCAACAACTTA			
		Eo_atp6_Probe		CCCCATAGTGTTGATTTTGCGTCCTTTCA	CY5-TAMRA		
*Taenia solium*	*NADH dehydrogenase* subunit iV	Tsol_nad4_Fwd	AB086256 (China) [27]	CCCAAAAACGGAACGACAA		106	This study
		Tsol_nad4_Rev		GCTGTGCATTATCTGTATCTTTTTTAATTG			
		Tsol_nad4_Probe		ATGCTAATCAACGCTTCCCATCTAACTCGC	HEX-TAMRA		
*Toxocara* spp.	*Cytochrome oxidase* subunit I	Toxo_spp_cox1_Fwd	AM411108 (for *T. canis*) (China) [23]	AAAATAGCCAARTCCACWCTMCTACCA		79	This study
		Toxo_spp_cox1_Rev	AM411622 (for *T. cati*) (China) [23]	GGTGTGGKACTAGTTGAACTGTGTA			
		Toxo_spp_cox1_Probe		CCCCATAGTCCTCAAAG	FAM-MGB		
Alea – PCR inhibior test	Random sequence	Alea_Fwd	[21]	CCTAAAAATGTCTATGATTGGTCCACTA		167	Knapp et al. [21]
		Alea_Rev		GGGAGTACCTTGCCATACAAAATT			
		Alea_probe		TTAAATCAACTCCTAAATCCGCGCGATAGG	VIC-TAMRA		

The PCR assays for differential diagnosis were designed to also amplify the DNA of other parasites likely to be encountered in the laboratory, such as *Taenia solium* and *Toxocara* spp. The *T. solium* PCR assay was designed in the present study, whereas that for *Toxocara* spp. was modified from a previous PCR assay designed for *T. cati* [21]. The present qPCR assay allows amplification of both *T. cati* and *T. canis* DNA because of degenerate nucleotides included in the primers (Table 2).

Each PCR assay was performed in a final volume of 20 μL containing 10 μL 2X TaqMan Gene Expression master mix (Life Technologies, Foster City, CA, USA), 5 pmol of each primer, 0.4 pmol of the hydrolysis probes with compatible fluorochromes, and 1 μL total DNA extracted from the studied specimens. Three multiplex PCR assays were designed to combine DNA detection of (1) *E. multilocularis*, *E. granulosus* s.s., and the external control (Alea) (MLX qPCR Em–Eg–Alea) and (2) *E. canadensis*, *E. ortleppi*, and Alea (MLX qPCR Ec–Eo–Alea), in combination or not with that of (3) *T. solium* and *Toxocara* spp. (MLX qPCR Tsol–Toxo).

Depending on the epidemiological and clinical context, MLX qPCR assays 1, 2, and 3 can be performed independently. The qPCR was run on a QuantStudio 5 real-time PCR model system (Life Technologies, Foster City, CA, USA). The PCR program was comprised of three steps: a first step at 50 °C for 2 min, a second step with denaturation at 95 °C for 10 min, and a third step with 45 cycles of denaturation at 95 °C for 15 s, followed by annealing and elongation at 60 °C for 1 min. In order to permit the late amplifications around 40 cycles to be complete, the number of 45 cycles was applied. All PCRs were performed in duplicate and the results are expressed as the mean of the quantitative cycle (Cq) number. A qPCR assay was considered positive when at least one reaction of the duplicate provided a positive result (Cq < 45 cycles).

### Real-time quantitative PCR design for differential diagnosis

#### Sensitivity of the assays

A dilution series was prepared to obtain a DNA range with eight concentration points to test the method detection limits (MDLs) or the probability of successfully detecting n positive results out of N trials for each qPCR assay. The technical limits of each individual qPCR assay were tested for each target by performing the PCR seven times on each of the eight points of the DNA concentration range. The last point of the DNA range giving 7/7 positive PCR trials was considered to be the MDL for the targeted DNA, meaning that the DNA was quantifiable under this threshold, whereas detection beyond the limit indicated that DNA was detectable but not quantifiable [17]. To perform the MDL tests, DNA from adult worms for *E. multilocularis* (15 ng/μL of DNA concentration), *T. cati*, and *T. canis*, provided by the CERFE laboratory (Boult-aux-Bois, France), was used (4 and 3 ng/μL of DNA, respectively), as well as the laminated layer from metacestodes isolated from animal lesions for *E. granulosus* s.s. (Anses code 5872, 11 ng/μL), *E. ortleppi* (Anses code 6697, 46 ng/μL) [38], and *E. canadensis* (Anses code 3734, 5 ng/μL) [39], provided by the ANSES laboratory (Malzéville, France), and the cysticercus stage of *Taenia solium*, provided by the Pasteur Institute (Paris, France) (3 ng/μL). The MDL tests were performed for each individual qPCR in duplex assays and included the external control Alea.

### Specificity of the assays

The generated primers were first tested for specificity using the NCBI Primer-BLAST tool (http://www.ncbi.nlm.nih.gov/tools/primer-blast/), which used the NCBI sequence databases for sequence alignment [42]. A mismatch threshold to ignore targets was applied from 6 differences. Second, a panel of samples (Table 1) or parasite DNA obtained from larval or adult specimens (for *T. solium*, *T. cati*, and *T. canis*) was tested for all qPCR assays to detect putative cross-reactions. These amplification reactions were performed in simplex PCR and in duplicate.

## Results

### Sensitivity of the assays

We assessed the sensitivity of the assays, which is presented through the MDL results (Table 3). The MDL for *Echinococcus* spp. (37–42 cycles), *T. solium* (37 cycles), *T. cati* (36 cycles), and *T. canis* (38 cycles) corresponds to the detection in 1 μL of the tested samples of 0.6 pg to 1 fg of DNA for *Echinococcus* spp., 0.3 fg for *T. solium*, 1 pg for *T. cati*, and 0.3 fg for *T. canis*. Standard curves were plotted for each PCR assay based on seven positive replicates from the DNA concentration range to obtain the slope and *Y*-intercept (from three to five dilution points with seven positive results used, Table 3). The efficiency of the TaqMan qPCR assays on DNA dilutions ranged from 84.6% to 136.9%.

**Table 3:** Method detection limits performed for each targeted parasite, with DNA dilution series tested on seven PCR assays.

Species	Last dilution 7 PCR(+) (ng/μL)	Mean Cq MDL (cycle)	SD	*Y*-inter	Slope	*r* ^2^	Efficiency (%)	*n* dilutions
*E. multilocularis*	5 × 10^−4^	37.97	0.84	27.37	−3.18	0.97	106.5	4
*E. granulosus* s.s.	1 × 10^−4^	42.21	1.00	28.76	−3.27	0.99	102.3	4
*E. canadensis*	5 × 10^−4^	36.82	0.87	26.20	−3.36	0.99	98.3	3
*E. ortleppi*	5 × 10^−4^	41.00	1.46	29.40	−2.67	0.99	136.9	3
*Taenia solium*	3 × 10^−5^	37.35	0.48	22.27	−3.31	0.99	100.5	4
*Toxocara cati*	4 × 10^−4^	36.15	0.78	23.71	−3.76	0.93	84.6	4
*Toxocara canis*	3 × 10^−5^	38.55	0.58	21.89	−3.55	0.99	91.2	5

Cq, cycle threshold; MDL, method detection limit; SD, standard deviation; *Y*-inter, *Y*-intercept; *r*^2^, correlation coefficient.

% Efficiency calculated by qPCR efficiency calculator available on ThermoFisher website.

*n* dilutions: number of dilution points with 7 positive qPCR used to calculate the standard curve plot.

### Specificity of the assays

The test of specificity performed using the online NCBI Primer-BLAST software showed good matching between designed primer sequences and referenced data for *E. multilocularis* (exact matching with 94 reference sequences versus 16 with one mismatch), *E. granulosus* s.s. (exact matching with three reference sequences versus two with one mismatch), *E. ortleppi* (exact matching with seven reference sequences), *Taenia solium* (exact matching with one reference sequence versus three with two mismatches), *Toxocara* spp. (for *T. cati*, exact matching with one reference sequence versus one with one mismatch and *T. canis*, exact matching with eight reference sequences versus one with two mismatches) and *E. canadensis* (exact matching with 127 reference sequences from the G6, G7 and G10 genotypes versus two mismatches with the G8 genotype). According to the Primer-BLAST tool, the current primer pair and probe designed for *E. canadensis* should correctly amplify at least the G6, G7 and G10 genotypes.

Specificity was tested on 50 DNA samples from echinococcosis lesions (21 AE and 29 CE samples) and DNA from the larval and adult specimens used for the sensitivity assessment (Table 1). The specificity was 100% for each qPCR assay on fresh and FFPE tissues from patients, as well as for larval and adult specimens from animals.

### Tested samples and the presence of PCR inhibitors

All 81 samples were analysed using one or three MLX qPCR assays. Each DNA sample was amplified by the appropriate specific assay, in agreement with the previous molecular or pathology-based identification (Table 1).

For the 34 fresh samples, *E. multilocularis* was detected by the specific qPCR assay with a mean Cq of 27.0 (95% CI 25.2–28.7), ranging from 23.7 to 36 cycles (14 ng to 2 pg of DNA detected, respectively). *Echinococcus granulosus* s.s. was detected by the specific qPCR assay with a mean Cq of 25.7 (95% CI 22.8–28.7), ranging from 16.0 to 33.2 cycles (8.0 μg to 10 pg of DNA detected, respectively). *Echinococcus canadensis* was detected by the specific qPCR assay with a mean Cq of 22.7 (95% CI 12.9–32.5), ranging from 14.7 to 28.2 cycles (2.6 μg to 0.2 ng of DNA detected, respectively). From the qPCR inhibitor test, one CE sample showed the presence of PCR inhibitors, but was positive for the qPCR Eg cox3 (Cq = 16). For the other samples the mean Alea Cq was 34.7 cycles (95% CI 32.9–33.8).

From among the 47 FFPE samples, 45 were amplified (Table 1). *Echinococcus multilocularis* was detected by the specific qPCR assay with a mean Cq of 31.8 (95% CI 30.1–33.5), ranging from 22.9 to 39.2 cycles (25 ng to 0.2 pg of DNA detected, respectively). *Echinococcus granulosus* s.s. was detected by the specific qPCR assay with a mean Cq of 35.4 (95% CI 33.1–37.7), ranging from 27.1 to 39.9 cycles (3 ng and 0.4 pg of DNA). *Echinococcus ortleppi* was detected by the specific qPCR assay with a mean Cq of 25.8 and 34.7 cycles, (59.4 and 0.03 ng of DNA detected respectively). Two AE samples from 2011 to 2013 were not amplified and showed a Cq of approximately 41 cycles in the previous study with the rrn qPCR applied to 5 μL of extracted DNA, the other PCR techniques being negative. One CE sample presented in the previous study was not amplified and showed only a positive result for the multiplex PCR developed by Trachsel et al. [37] in the previous study. The mean Alea Cq was 34.7 (95% CI 34.4–35.0).

A Welch *t*-test was performed on the parasite qPCR Cq values obtained between fresh and FFPE samples, highlighting a significant difference (*p* < 0.001). FFPE samples with molecular confirmation first (MB) were sampled between 2012 and 2021 and FFPE samples with a pathology exam first (PATH) were sampled between 1997 and 2016. A Welch *t*-test was performed on the parasite qPCR Cq values obtained between the two groups and showed a significant difference (*p* = 0.027), with Cq values lower in samples with molecular confirmation first, being isolated more recently.

## Discussion

*Echinococcus* species typing is a key point in the diagnosis of echinococcosis in humans to differentiate alveolar from cystic echinococcosis infections, which is essential for patient management and treatment. In this study, we developed an MLX qPCR assay able to detect the four species mainly involved in echinococcosis in Europe, and we showed that the high sensitivity of the technique enables testing with very small amounts of DNA. The MLX qPCR assay is also particularly helpful for identifying degraded DNA in paraffin-embedded samples.

In the present study, specificity was 100% for all targets, without cross-reaction between primer-pair couples used for the various parasite species. However, even though the number of *E. multilocularis* and *E. granulosus* s.s. specimens was adequate to test the specificity and sensitivity of the MLX qPCR technique, a more extensive sampling effort has to be made to conclude about performance of this assay for the detection of *E. ortleppi* and *E. canadensis*. In fact, only four fresh samples from *E. canadensis* and two FFPE from *E. ortleppi* were analysed in this study.

The MLX qPCR assays developed here show high sensitivity and allow for the detection of *Echinococcus* spp. that infect humans in Europe using a small volume of DNA extract. Due to the design of the primers and probes, targeting a small fragment of mitochondrial DNA, the assay is particularly efficient for diagnosis from FPPE, where DNA is fragmented. However, the qPCR failed for three FFPE samples. Further assays using higher DNA volumes were not attempted because of the lack of available left-DNA sample. Moreover, these samples, for which the DNA was extracted >5 years after paraffin inclusion could have become highly degraded during storage, as previously described [14, 19], and could hinder echinococcosis diagnosis, especially in cases of diagnostic wandering. An association with other PCR techniques may be necessary in such particular cases with degraded DNA, such as the multiplex PCR assay described by Trachsel et al. [37] and the Em-rrn qPCR assay used with success on old samples [19].

A control for the presence of PCR inhibitors was integrated into the multiplex qPCR assays, allowing the validation of negative results. However, with high DNA concentrations, the Alea qPCR amplification can be negative, as observed in 1/56 routine samples. This could be due to consumption of the Taq polymerase during the first cycles of the qPCR for the parasite DNA amplification or the presence of inhibitors, without an impact on target parasite qPCR.

The MDL tests were performed on pure parasite DNA or with a minimum of contamination by the host DNA host. Importantly, contamination with human DNA occurs, especially in cases of AE lesions, because of the significant infiltration of the parasite into the intermediate host tissue. The DNA concentration values measured before the qPCR, e.g., by spectrophotometry, are not always informative. The sensitivity of the *Echinococcus* spp. PCR is below the picogram range of DNA for seven positive PCRs, providing the limit of parasite quantification in the tested samples. Beyond this limit, the parasite can be detected but not quantified. Moreover, the MDL can permit us to take a critical view on late amplifications, and if applicable, repeat the analyses to avoid false-positive results. We chose to apply 45 cycles to the MLX qPCR in order to permit late amplifications around 40 cycles to be completed. From the MDL tests, e.g. for *E. granulosus* s.s., the seven PCR tests remained positive on the DNA concentration of 1 × 10^−4^ ng/μL after 42 cycles of PCR. As for other infectious diseases, the positive threshold can be up to 40 cycles (example 43 cycles for *Aspergillus* spp. PCR) [41]. From quantitative results, we found significant differences based on the type of tissue conservation, i.e., fresh versus FFPE tissues, with lower Cq values obtained by qPCR for fresh than FFPE tissues, as previously observed for end-point PCR [12]. We also observed a significant difference between recent and older FFPE samples. The storage time of FFPE samples is critical for PCR results, as previously observed, with a large decrease in PCR performance after four to five years of storage [14, 19]. Moreover, the sensitivity of the technique on FFPE samples may be linked to the macroscopic selection of the parasitic zone followed by the extraction of DNA from this zone, as in the estimation of the percentage of tumour cells presenting mutations in pathology examinations [25]. Concerning the DNA panel tested, the samples can be considered to be representative of specimens received for routine analysis in terms of the quality and concentration of parasite DNA and the presence of host DNA. Diagnosis can be made on FFPE samples included in paraffin several months or years before the analysis (data from the National Reference Centre for Echinococcoses).

The high sensitivity of the presented assays could permit the detection of parasites in other matrices, such as blood, cerebrospinal fluid, or even urine. Cell-free circulating DNA could be targeted, especially as the techniques for the diagnosis of both alveolar and cystic echinococcosis still need to be improved [2, 10, 18, 24, 36, 44] and replaced with newer approaches based on non-invasive, sensitive assays, such as those permitted by real-time qPCR.

The detection of early infections using molecular tools could allow for more efficient patient care and access to radical surgery, thus reducing the time of chemical treatment, reducing complications due to diagnostic wandering, and increasing patient quality of life. Moreover, in certain cases, imagery and serology as first-line diagnosis may not be conclusive, as observed for immunosuppressed patients [6], with co-morbidities making the diagnosis all the more difficult. Moreover, in cases of echinococcosis due to species other than *E. multilocularis* and *E. granulosus* s.s., for which the commercial immunodiagnostic kits are specifically designed, the diagnosis is even more difficult. Among the *E. granulosus* s.l. complex, *E. granulosus* s.s. associated with a dog-sheep life cycle is the most important cause of cystic echinococcosis in the world [1, 5]. However, infection by other species, such as *E. ortleppi* described in France [3, 13] and *E. canadensis* has been described (11% of CE due to *E. canadensis* G6–G7 [1]). A rapid screening test for multiple targets would be an efficient solution in cases of diagnostic wandering or the misidentification of the parasite species.

Here, we targeted cysticercosis and toxocariasis to help in the context of wandering diagnosis, as such molecular diagnosis is occasionally requested for some patients with atypical lesions (e.g. liver abscess either described in echinococcosis or toxocariasis) for immunosuppressed patients. However, no positive clinical samples were available in the present study, and only the theoretical limits of detection and the specificity could be assessed. Nevertheless, the qPCR assays targeting these species are now available for laboratories; the development of the protocol was made for DNA isolated from parasites (adult stage or isolated cyst, that were not or only slightly contaminated by host DNA), and additional clinical samples are needed for complete validation and evaluation in the clinical setting.

## Conclusion

The present work proposes a sensitive multi-target diagnosis on four *Echinococcus* species occurring in Europe, with PCR inhibitor control, and a substantial gain in sensitivity for FFPE specimens. The present molecular assays can be applied as an accurate screening technique in the context of clinical evaluation and imaging studies for a suspected echinococcosis infection, as well as for atypical forms and in differential diagnoses to rule out other parasites.
